# Determination of Metals in Tree Rings by ICP-MS Using Ash from a Direct Mercury Analyzer

**DOI:** 10.3390/molecules25092126

**Published:** 2020-05-01

**Authors:** Byunggwon Jeon, James V. Cizdziel

**Affiliations:** Department of Chemistry and Biochemistry, University of Mississippi, University, MS 38677, USA; bjeon@go.olemiss.edu

**Keywords:** mercury pollution, tree rings, trace elements, ashing, national forest, direct mercury analyzer, ICP-MS

## Abstract

Elemental profiles in cores of tree trunks (bole wood) have been used for environmental monitoring and reconstruction of metal pollution history. Mercury (Hg) is a global pollutant that can be accurately measured in tree rings in a simple and pragmatic fashion using a direct mercury analyzer (DMA) that is based on thermal decomposition, amalgamation, and atomic absorption spectrophotometry. In this feasibility study, we demonstrate that the ash remaining after the DMA analyses can be used to quantify a wide range of other non-volatile elements (Ba, Be, Co, Cr, Cu, Fe, Ga, Mg, Mn, Ni, Pb, Sr, Th, and U) in that same sample of wood by inductively coupled plasma mass spectrometry (ICP-MS) after microwave-assisted acid digestion. Other elements (Ag, Cd, Cs, Rb, Tl, and V) exhibited poor recoveries, possibly due to losses during sample preparation. We assessed the accuracy with reference materials, spikes, and by comparison with EPA Method 3052 (Microwave Assisted Acid Digestion of Siliceous and Organically Based Matrices). For the first group of elements (deemed suitable for the method), recoveries ranged between 80% and 120% and the relative standard deviation was generally < 15%, indicating acceptable precision. We applied the method to five species of trees: eastern red cedar (*Juniperus virginiana*), loblolly pine (*Pinus taeda*), shortleaf pine (*Pinus echinata*), white oak (*Quercus alba*), and tulip poplar (*Liriodendron tulipifera*) from Holly Springs National Forest in north Mississippi, USA. Mercury concentrations (ng/g ± SE) were highest in the cedar (1.8 ± 0.3; *n* = 5), followed by loblolly pine (1.6 ± 0.3, *n* = 3), shortleaf pine (1.2 ± 0.2; *n* = 3), oak (1.1 ± 0.2; *n* = 5), and poplar (0.5 ± 0.1; *n* = 5). Concentrations of other elements were generally Fe > Mg > Ba ≈ Sr ≈ Mn > Cr ≈ Cu > Ni ≈ Rb > Co > Ga ≈ Ag, with the other elements generally below the method detection limit (MDL). Overall, we showed that the DMA can be used to not only determine total Hg in segments of tree core, but can serve as the ashing step in the preparation of wood for ICP-MS analysis, thus allowing the determination of non-volatile elements along with Hg in the very same sample.

## 1. Introduction

Profiles of trace elements, including Hg and other toxic metals, in tree rings have been used in a variety of studies worldwide for environmental monitoring and the reconstruction of pollution history [1,2,3,4]. These dendrochemical investigations rely on the preservation of a chemical record of metal deposition from the local environment incorporated into the annual growth increments of trees [5]. In some cases, when there are overlapping features from living and dead trees that have preserved timber, annually resolved chronologies can stretch back hundreds of years [6]. However, using tree rings as records of environmental pollution requires caution due to potential differences in both the mobility of elements in wood and in physiological mechanisms of uptake of elements among species [7]. Whereas analysis of an individual tree-ring may not pinpoint a specific year of environmental change, trees can record long-term patterns and serve as biomonitors of macro-environmental trends [8]. Thus, there continues to be interest in rapid and pragmatic analytical methods for dendroanalysis, especially for methods capable of determining multiple elements, including Hg.

Mercury is a global pollutant transported through the atmosphere that is incorporated into tree tissues primarily through atmospheric deposition [4,9,10]. Monitoring Hg in the environment is essential for evaluating the effectiveness of the recent (2017) Minamata convention, a global regulatory mechanism to decrease environmental Hg loadings [11]. While some studies have shown that tree cores can serve as temporal total-Hg biomonitors, recording changes in Hg deposition with pollution history [4,10,12], others have not [13], suggesting that the cycling of Hg in individual species of trees needs to be further investigated. In any case, quantifying Hg in tree wood and other tissues is also important to understanding the pools and fluxes of Hg in forest ecosystems [10]. However, measuring Hg in trees, especially wood, is difficult due to its low concentration, which is often below method detection limits [14]. Despite its low concentration, the Hg content of wood often comprises the largest pool of Hg in forests, except for the soil, due to its relatively high biomass [15]. Moreover, wildfires are a poorly characterized but potentially important source of Hg to the atmosphere, and a lack of data on Hg in wood is leading to uncertainty in ecosystem Hg budgets [10,14].

Recently, Yang et al. [14] showed that a direct mercury analyzer (DMA), based on thermal decomposition, catalytic conversion, amalgamation, and atomic absorption spectrophotometry, can reliably measure Hg in wood. The method is popular because compared to inductively coupled plasma mass spectrometry (ICP-MS), it is much less costly, requires virtually no sample preparation, is rapid (<6 min/sample), and can be run by students after minimal training. Moreover, whereas Hg can be effectively determined by ICP-MS, the analyst must be keenly aware of a number of pitfalls, including potential carryover as Hg is prone to “memory” effects (e.g., sorption/desorption from pump tubing). Mercury also has a relatively high ionization potential (~10.4 eV) and its signal is spread out over its seven stable isotopes. Thus, Hg in bole wood is increasingly being determined with DMAs. However, the ash that remains after the Hg analysis is typically discarded, despite containing non-volatile elements whose concentration can also be determined for pollution and other dendrochemistry-related studies.

The purpose of the current study was to develop and validate an analytical method for the analysis of bole wood tree rings which combines the determination of total-Hg using the DMA with multi-elemental analysis using ICP-MS. Effectively, we used the DMA as a sample-ashing step before microwave-assisted acid digestion and ICP-MS analysis of the diluted bole wood digests. As we have not seen this approach documented in the scientific literature, and because others are now routinely measuring Hg in tree wood using DMAs as it has becomes apparent that wood is, by mass, the most important reservoir of Hg in forests after soil [16,17], we carefully evaluate and optimize the method. Here, we report on the methodology and its application to trees from Holly Springs National Forest in Mississippi, which will serve as a background site for future dendrochemical studies tracing the pollution history of coal-fired power plants and other potential point sources of airborne metal pollution in the region. We focused on the outer (most recent) tree rings; fully quantifying the distribution and trends of Hg and other elements in the full tree cores is beyond the scope of this method feasibility study.

## 2. Results and Discussion

### 2.1. Method Comparison, Figures-of-Merit, and Recovery Tests

EPA method 3052 is a common microwave digestion method applicable to organic matrices that uses 9 mL of concentrated HNO_3_ and 3 mL of concentrated HF. However, given our sample size (~0.1 g) and the low concentration of many elements in wood, we modified the method to reduce dilution while maintaining optimal digestion conditions. To compare our DMA-ICPMS method to the EPA method, we prepared a homogenized mixture of pulverized bole wood from white oak (*Quercus alba*) by cryogenic grinding (Spex 6875 Freezer/Mill) of the wood from multiple tree cores. The material was analyzed directly by EPA method 3052 and by our method after ashing with a DMA. Our method, described later, uses three “micro-inserts” in each digestion vessel and only 1.5 mL HNO_3_ and 50 µL of HF. The results show good agreement between the two approaches, except for Cd, Cs, and Tl (Table 1). Cadmium is known to be volatile and is likely lost during the DMA analyses; Cs can form a metal oxide that can attack silica, and Tl was near the MDL (Table 1).

To estimate the MDL and limit of quantitation (LOQ) (3σ and 10σ criteria, respectively), multiple reagent blanks were included with each set of digestions. MDLs ranged from <5 ng/g for Ag, Co, Cs, Ga, Th, Tl, U, V to 1.2 µg/g for Mg (Table 1). All calibration curves were >0.994. For those elements suitable for the method, the relative standard deviation for the reference material was generally <15%, indicating acceptable precision.

To check the accuracy of the method, we also analyzed reference materials, including CRM AR 1946 (wood fuel biomass) and NIST SRM 1633C (coal fly ash), the latter with and without DMA analysis (ashing). In addition, because only three of the twenty elements determined were certified in the wood reference material (six others had reference values), we spiked the wood CRM with 10 µL of a 10 µg/mL multi-element standard (to yield ~0.4 ng/g in the final diluted solution) and analyzed the material both with and without DMA ashing using microwave digestion and ICP-MS. The results for these recovery tests are summarized in Table 2. Recoveries for the three certified elements, Mg, Fe, and Mn, in the wood reference material were 93%, 99%, and 110%, respectively. Recoveries for Sr (97%), Ba (117%), and Cu (118%) were also acceptable. Recoveries for Ni, Pb, and V were low (31% to 51%); however, these elements only had reference values and Ni and Pb performed much better with spikes of the wood CRM as well as the other reference material where their concentration was certified. Recoveries for V were mixed with some low values ~60%; thus, the element should be carefully monitored as it may not be suited for this method.

Recoveries for the coal fly ash ranged from 80% to 110% for Ba, Co, Cr, Cu, Mn, Ni, Pb, Sr, U, V for both DMA-heated samples and samples that were analyzed directly without the DMA. Recoveries for Cd, Cs, Tl were poor, as was the case in our comparison with the EPA method (above), suggesting that these elements are also not well-suited for this method. Recoveries from the coal fly ash were also poor for Fe, Mg, Th, and Rb, but that was the case for both DMA and non-DMA samples and given good recoveries from the wood reference material (both with and without DMA-ashing), the effect is likely matrix-dependent and coal fly ash is substantially different from wood ash. Thus, we feel that the method is suitable to determine Fe, Mg, Th and Rb in tree cores.

In summary, Ag, Cd, Cs, Rb, Tl, and V are problematic and probably should not be quantified using this method; the remaining elements (Ba, Be, Co, Cr, Cu, Fe, Ga, Hg, Mg, Mn, Ni, Pb, Sr, Th, and U) can be quantified if the concentration is above the MDL in the tree wood. In any case, we emphasize that it is important that each laboratory documents the reliability of their own data using appropriate quality assurance measures.

### 2.2. Analysis of Tree Cores Collected from the Holly Springs National Forest

**Figure 1:**
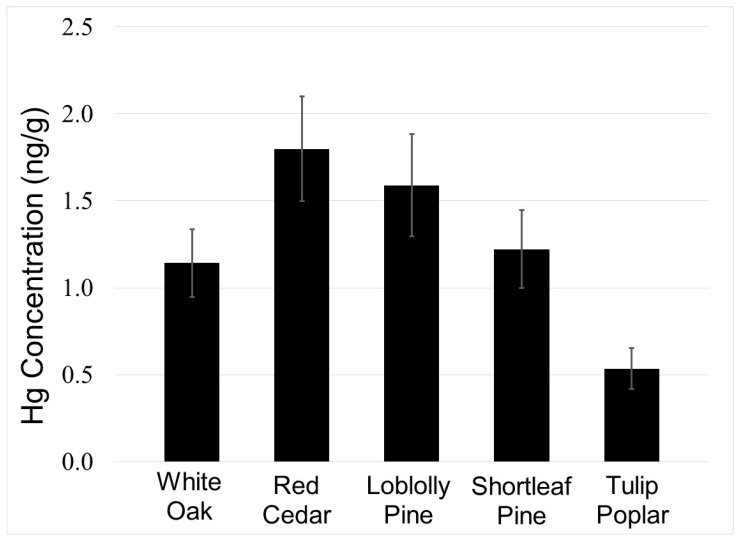
Mean mercury concentration in tree rings from the Tallahatchie Experimental Forest in Holly Springs National Forest in north Mississippi determined with a direct mercury analyzer. Error bars are ± 1 standard error.

We applied the method to bole wood collected from five tree species in Holly Springs National Forest. Mercury concentrations (ng/g ± 1 SE) were highest in the cedar (1.8 ± 0.3; *n* = 5), followed by loblolly pine (1.6 ± 0.3, *n* = 3), shortleaf pine (1.2 ± 0.3; *n* = 3), white oak (1.1 ± 0.2; *n* = 5), and tulip poplar (0.5 ± 0.1, *n* = 5) (Figure 1). These concentrations are similar with those reported in other studies of tree cores: for example, Hg concentrations in cores deciduous trees cores from Ontario, Canada, including poplar (*Populus deltoids*), willow (*Salix x rubens*), and red oak (*Quercus rubra*) ranged from ~0.7 to 4.1 ng/g [13], and in hardwoods and conifers from the northeast USA, American beech (*Fagus Grandifolia*), yellow birch (*Betula Alleghaniensis*), red maple (*Acer Rubrum*), sugar maple (*Acer Saccharum*), red spruce (*Picea Rubens*), white ash (*Fraxinus Americana*), white pine (*Pinus Strobus*) and balsam fir (*Abies Balsamea*) ranged from ~0.4 to 5 ng/g [10,13]. It is important to note that recent work has shown the suitability of tree rings as archives of global and regional atmospheric Hg pollution [18], and has been used track atmospheric Hg pollution, including from past mining operations in Australia [19].

**Figure 2:**
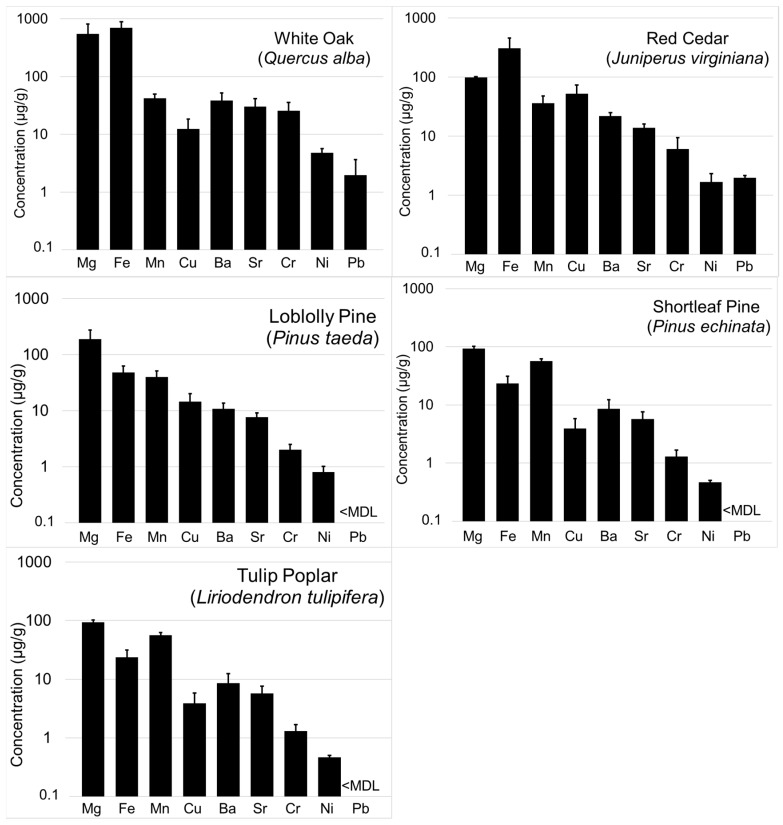
Mean concentrations of elements in bole wood tree rings from the Tallahatchie Experimental Forest in Holly Springs National Forest in north Mississippi determined by ICP-MS using ash from a direct mercury analyzer. Note: *y*-axis is on a logarithmic scale and error bars are ± 1 standard error.

Concentrations of elements determined in the DMA-ash by ICP-MS were generally in the following order, Fe > Mg > Ba ≈ Sr ≈ Mn > Cr ≈ Cu > Ni > Co > Pb, with several other elements (Be, Th, Ga, U) routinely near or below the MDL regardless of species (Figure 2, Table 3). In general, elemental concentrations were higher in the slow-growing oak and cedar, compared to the pine and tulip poplar. Most of the trace metal concentrations are comparable to measurements of tree rings from other studies conducted in relatively pristine areas, and lower than those from contaminated areas. Cocozza et al. [20] measured relatively high concentrations of Co (>50 µg/g) in tree rings collected from downy oak (*Quercus Pubescens*) near an incinerator in an industrial area. Our highest measurement of Co was 0.41 µg/g. In another study, Madejón et al. [21] measured 63 µg/g of Pb in white poplar (*Populus Alba*) in riparian forests contaminated from a mine waste spill. All but one of our measurements for Pb were <1 µg/g.

Overall, in this communication, we demonstrate that DMAs based on combustion atomic absorption spectrophotometry can be used effectively as an ashing step in the preparation of tree cores for ICP-MS analysis for certain metals, which allows the determination of non-volatile elements in that very same sample. Finally, we note that continued advancements in ICP-MS, including collision/reaction cell- or laser ablation-ICP-MS, may make the technique more suitable for direct multi-element analyses (including mercury) in wood samples.

## 3. Materials and Methods

### 3.1. Site Description and Collection of Tree Cores

**Figure 3:**
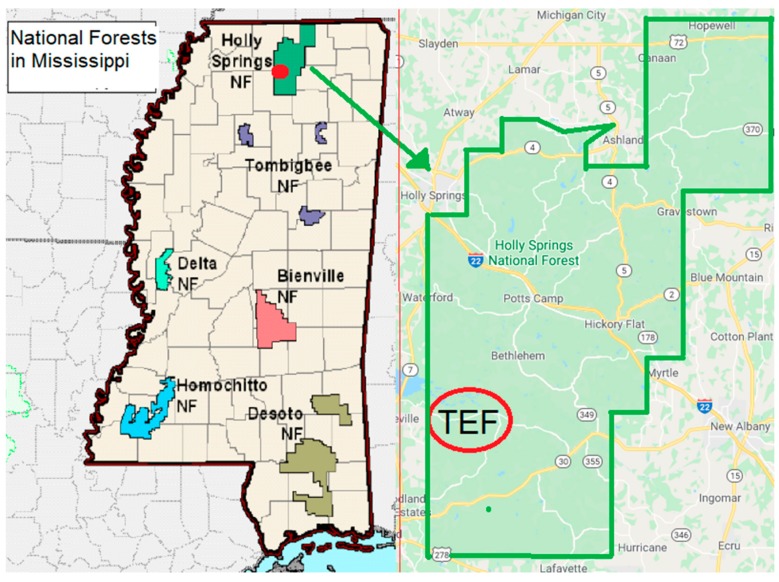
Location of the Tallahatchie Experimental Forest (TEF) in Holly Springs National Forest (NF) in north central Mississippi, USA.

Tree cores were collected from five species of tree—eastern red cedar (*Juniperus virginiana*), loblolly pine (*Pinus taeda*), shortleaf pine (*Pinus echinata*), white oak (*Quercus alba*), and tulip poplar (*Liriodendron tulipifera*)—from Tallahatchie Experimental Forest (TEF), which is part of the Holly Springs National Forest located in the north central hills of Mississippi (Figure 3). The forest encompasses 629 km^2^ and was established in 1936 after loblolly pine was planted in the area to convert rapidly eroding abandoned agricultural land to forest. The TEF, located near Oxford, Mississippi, was created in 1950 to study relationships between mixed pine and hardwood forests, flooding, and soil erosion.

**Figure 4:**
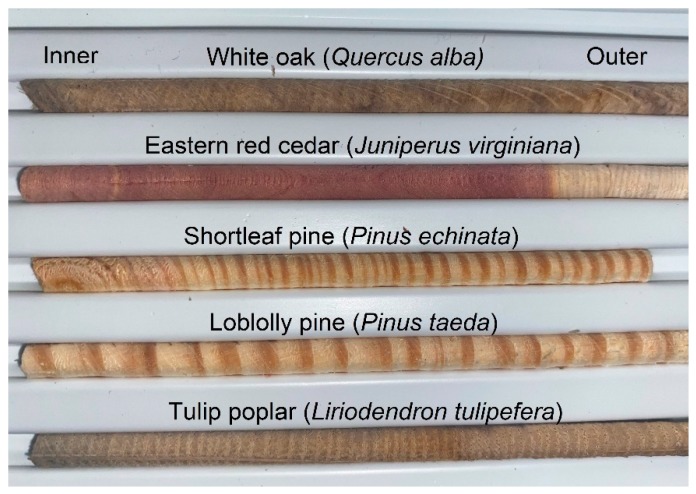
Example of tree cores collected with a 5-mm increment borer.

Trees were identified using bark and leaves, and cores were collected at about 1 m above the ground using a 5.15-mm diameter increment borer made from hardened steel with a PTFE coating (Haglöf, Sweden) or an increment hammer, also made of steel (Figure 4). The extractor is stainless steel. All tools were cleaned and rinsed with deionized water and methanol before sampling. The tools were inspected between uses to make sure no material was transferred between samples. Given the Teflon coating and hardened materials used, we do not expect any impact on metal concentration in the wood. Extracted cores were inserted in an increment core holder, which was placed into Ziploc bags for transport. The cores were air-dried in a laminar flow hood in a clean room for ~1 week before being stored in a desiccator. This was found to be sufficient for complete dissication before analysis.

### 3.2. Direct Mercury Analysis

Total Hg was determined in ~1 cm (~0.1 g) segments of tree core using a DMA-80 (Milestone Inc., Shelton, CT, USA) following EPA Method 7473 with slight modifications. Briefly, the bole wood was weighed into quartz boats and the boats placed in the instrument’s autosampler. The boats were sequentially inserted into the unit’s combustion tube where the sample was thermally decomposed (combusted) at 650 °C with air serving as the carrier gas (200 mL/min). Instrument parameters are given in Table 4. The total analysis time was < 5 min per sample. The instrument was calibrated from 0.05 to 100 ng of Hg using six standards prepared from 10 µg/mL Hg standard solution (Spex Certiprep, Metuchen, NJ, USA). The system was checked for accuracy at the beginning and end of each run using SRM1633C (Coal Fly Ash). Recoveries were between 90% and 115%. Ash residue was carefully weighed into micro-sampling inserts for microwave digestion.

### 3.3. Microwave-Assisted Acid Digestion of Tree Core Ash

A general scheme showing the analytical process is given in the graphical abstract. The ash remaining after DMA analysis of the dried tree core segments (~10 mg) was digested with 1.5 mL of HNO_3_ and 50 µL of HF (both trace metal grade, Fisher Chemical, Pittsburgh, PA, USA) in acid-cleaned 6 mL TFM micro sampling inserts using a 1200 W Ethos microwave digestion system (Milestone Inc., Shelton, CT, USA). The temperature program consisted of a 15 min ramp to 200 °C and a 20 min hold at that temperature. The resulting digest was transferred to polyethylene tubes and diluted to 50 mL with ultrapure water (deionized and 0.2 µm-filtered with a Milli-Q system; Millipore, Burlington, MA, USA). An additional 1:5 dilution was carried out in 2% HNO_3_. Sc, Rh, and Bi were used as internal standards at a final concentration of 1 ng/mL. Note, further dilution may be needed for certain elements like Mg and Ba depending on instrument sensitivity and concentrations in the samples. Reagent blanks were run with every set of digestions. Two reference materials were used to validate the new method. AR1946 is a wood fuel biomass CRM (Alpha Resources LLC, Stevensville, MI, USA) and SRM1633C is a coal fly ash SRM (NIST; Gaithersburg, MD, USA). The digested solutions were analyzed using a sector field-ICP-MS.

### 3.4. Determination of Metals in Ash from the DMA by ICP-MS

Concentrations of 20 elements were determined by sector field ICP-MS using a Thermo Fisher Element-XR. The instrument features resolving power (m/Δm) settings of low (~300) and medium (~3000) to remove certain isobaric interferences. We measured Be, Rb, Sr, Ag, Cd, Cs, Ba, Tl, Pb, Th and U in low resolution and Mg, V, Cr, Mn, Fe, Co, Ni, Cu, and Ga in medium resolution. Data acquisition parameters are given Table 4. Samples were introduced using a PFA concentric nebulizer and a PFA cyclonic chamber (Elemental Scientific, USA). The system was optimized for sensitivity and stability, yielding ~0.8 million counts-per-second for 1 ng/g of ^115^In in low resolution mode with <3.5% relative standard deviation (RSD). The mass window was set to 20% with 50 points per peak for low resolution mode and to 125% with 20 points per peak for medium resolution mode. Elements were quantified using external calibration which consisted of a blank, 0.05, 0.1, 0.2, 0.5, 1, 5 ng/g standards from a multi-element solution (Spex CertiPrep, Metuchen, NJ, USA). The calibration curve correlation coefficient (r^2^) values were >0.994 for each element.

To back-calculate the concentration of the metals in the wood, the solution concentration was multiplied by a dilution factor consisting of the mass of the solution (after digestion and dilution to 50 mL mark) divided by the original weight of sample multiplied by 5 to account for the 1:5 dilution of the digest prior to analysis. Concentrations reported herein are based on dry-weight.

## Figures and Tables

**Table 1:** Comparison of the new method (with DMA ashing) with a standard method (EPA 3052, without DMA ashing) for determination of metals in pulverized bole wood prepared by cryogenic milling. Figures-of-merit are for the new method. Starred values indicate a relative percent difference (RPD) exceeding 25%, suggesting that the element may not be suitable for this method.

Element	Mean (±1 SD; *n* = 5)		RPD	MDL	LOQ	Calib. Curve Linearity (r^2^)
	EPA 3052	New Method				
Ag (ng/g)	11.5 ± 6.9	<MDL	-	5.1	17.2	0.9995
Ba (µg/g)	200 ± 8	192 ± 4	4.1	0.04	0.12	0.9990
Be (ng/g)	80 ± 10	82 ± 18	3.1	15.2	50.7	0.9996
Cd (ng/g)	250 ± 8	67 ± 12	73.3 *	7.2	23.9	0.9999
Co (ng/g)	348 ± 24	353 ± 47	1.4	1.5	4.9	0.9999
Cr (µg/g)	34.5 ± 4.4	31.0 ± 1.5	10.4	0.05	0.18	0.9999
Cs (ng/g)	6.0 ± 0.3	10.1 ± 4.4	67.2 *	2.1	7.1	0.9993
Cu (µg/g)	1.9 ± 1.0	1.7 ± 1.3	14.2	1.1	3.7	0.9999
Fe (µg/g)	156 ± 20	188 ± 56	20.8	0.81	2.72	0.9961
Ga (ng/g)	7.0 ± 2.5	8.6 ± 4.4	23.5	1.3	4.5	0.9999
Mg (µg/g)	1200 ± 48	1200 ± 71	1.6	1.2	3.9	0.9947
Mn (µg/g)	100.0 ± 4.6	96.3 ± 0.9	3.7	0.10	0.34	0.9986
Ni (µg/g)	13.4 ± 1.6	13.2 ± 0.5	1.4	0.05	0.17	0.9999
Pb (µg/g)	1.71 ± 0.07	1.72 ± 0.06	0.9	0.06	0.19	0.9998
Rb (µg/g)	4.52 ± 0.17	4.30 ± 0.06	4.8	0.02	0.08	0.9999
Sr (µg/g)	49.5 ± 2.1	47.0 ± 0.3	5.2	0.02	0.07	0.9999
Th (ng/g)	3.2 ± 4.3	<MDL	-	2.4	7.9	0.9994
Tl (ng/g)	4.7 ± 0.5	3.0 ± 0.7	36.4 *	2.9	9.8	0.9994
U (ng/g)	4.4 ± 2.3	3.6 ± 1.5	19.5	1.7	5.6	0.9986
V (ng/g)	210 ± 30	220 ± 20	0.9	3.2	10.5	0.9999

Note: Method detection limit (MDL) and limit of quantitation (LOQ) are based on a 0.1 g sample of wood.

**Table 2:** Recoveries for reference materials determined by ICP-MS after microwave digestion with or without prior ashing by a direct mercury analyzer.

Element (Unit)	CRM AR 1946 (Wood Fuel Biomass)					NIST SRM 1633C (Coal Fly Ash)								AR1946 Spiked with Multi-element Standard			
	Certified or Reference Value		With DMA Ashing (*n* = 3)			Certified or Reference Value		With DMA Ashing (*n* = 8)			Without DMA Ashing (*n* = 8)			With DMA Ashing (*n* = 2)		Without DMA Ashing (*n* = 3)	
	Mean	1 SD	Mean	1 SD	Rec. (%)	Mean	1 SD	Mean	1 SD	Rec. (%)	Mean	1 SD	Rec. (%)	Rec. (%)	RPD	Rec. (%)	SD
Ag (ng/g)	-	-	14.0	6.0	-	-	-	<MDL	0.1	-	<MDL	0.0	-	96.3	10.6	123.4	7.4
Ba (µg/g)	47 (ref)	-	54.9	2.0	116.8	1126	33	1158	83.6	102.9	1243	70.3	110.4	96.8	5.7	91.9	11.8
Be (ng/g)	-	-	<MDL	4.0	-	-	-	<MDL	-	-	<MDL	-	-	86.6	12.1	114.5	7.5
Cd (ng/g)	-	-	39.0	11.0	-	0.758	0.005	<MDL	-	156.4	<MDL	-	140.7	7.6	110.4	110.0	7.7
Co (µg/g)	-	-	<MDL	0.1	-	42.9	3.5	36.8	3.4	85.9	37.8	0.8	88.0	76.0	16.8	103.7	11.7
Cr (µg/g)	-	-	2.2	0.2	-	258 (ref)	6	207	18	80.2	217.8	2.1	84.4	98.2	4.9	123.0	18.1
Cs (ng/g)	-	-	35.0	6.0	-	9.39 (ref)	0.22	4.7	1.2	50.0	8.0	0.2	85.2	76.9	7.4	114.1	8.1
Cu (µg/g)	4 (ref)	-	4.7	3.0	118.3	173.7	6.4	154.8	12.0	89.1	159.3	3.4	91.7	-	-	100.1	14.9
Fe (%)	0.110	0.020	0.109	0.012	99.4	10.49	0.39	1.48	0.12	14.1	1.51	0.04	14.4	86.6	0.3	85.6	9.1
Ga (ng/g)	-	-	112.0	3.0	-	-	-	47.6	4.1	-	48.5	1.1	-	87.8	12.6	110.4	9.7
Mg (µg/g)	480	10.0	445	8.6	92.7	4980	520	278	81.5	14.0	637.5	26.4	32.2	93.0	6.4	87.4	14.8
Mn (µg/g)	110	10.0	121	3.7	109.8	240.2	3.4	204	21.7	84.8	207.7	3.5	86.5	94.2	8.2	90.0	14.1
Ni (µg/g)	3 (ref)	-	1.3	0.2	43.5	132.0	10.0	129	21.6	97.6	124.6	2.5	94.4	80.6	39.2	111.2	7.1
Pb (µg/g)	4 (ref)	-	1.2	0.3	31.1	95.2	2.5	95.1	5.3	100.1	91.0	1.4	95.6	97.4	100.2	111.1	0.8
Rb (µg/g)	-	-	2.9	0.0	-	117.42	0.53	31.2	2.2	26.6	43.8	2.4	37.3	56.4	68.3	95.7	15.6
Sr (µg/g)	33 (ref)	-	32.1	0.4	97.2	901	56	730	65	81.0	858.2	10.8	95.3	97.5	9.5	96.7	7.1
Th (ng/g)	-	-	82.0	13.0	-	23.0 (ref)	0.4	<MDL	-	7.4	3.8	0.1	16.5	83.9	9.8	109.1	18.8
Tl (ng/g)	-	-	<MDL	0.0	-	-	-	5.9	0.5	-	5.7	0.1	-	52.9	16.4	114.9	7.3
U (ng/g)	-	-	30.0	7.0	-	9.25 (ref)	0.45	8.4	0.6	90.8	8.0	0.1	86.5	85.1	13.8	117.0	8.3
V(ng/g)	3 (ref)	-	1.7	0.2	58.0	286.2	7.9	247	19	86.3	256.3	4.2	89.5	61.1	21.1	105.0	0.4

Note: Dashes (-) = not available; RPD = relative percent difference.

**Table 3:** Concentration of elements in tree cores from the Tallahatchie Experimental Forest in Holly Springs National Forest in north Mississippi determined by ICP-MS using ash from a direct mercury analyzer.

Species:	*Quercus Alba*			*Juniperus Virginiana*			*Pinus Taeda*			*Pinus Echinata*			*Liriodendron Tulipifera*		
	Tree 1	Tree 2	Tree 3	Tree 1	Tree 2	Tree 3	Tree 1	Tree 2	Tree 3	Tree 1	Tree 2	Tree 3	Tree 1	Tree 2	Tree 3
Circumference (cm):	137	129	80	57	48	47	32	88	153	88	109	83	151	109	160
Ag (ng/g)	<MDL	<MDL	<MDL	13.1	20.9	<MDL	<MDL	<MDL	<MDL	-	<MDL	<MDL	<MDL	<MDL	<MDL
Ba (µg/g)	26.4	66.4	23.2	27.4	21.9	17.1	16.2	9.0	7.0	6.5	15.9	3.2	19.1	49.4	29.3
Be (ng/g)	<MDL	41.3	<MDL	<MDL	<MDL	<MDL	<MDL	<MDL	<MDL	<MDL	<MDL	<MDL	<MDL	<MDL	<MDL
Co (ng/g)	196	414	237	93.9	48.9	141	62.7	58.1	20.5	32.2	35.6	15.4	42.5	-	89.8
Cr (µg/g)	22.7	10.6	44.1	4.8	1.1	12.5	1.6	3.0	1.4	1.2	2.0	0.7	1.6	3.3	3.5
Cu (µg/g)	2.3	12.6	22.5	83.4	62.2	13.2	25.1	12.7	5.4	2.3	7.7	1.7	3.0	6.1	7.1
Fe (µg/g)	687	373	1041	321	33.8	567	48.7	72.1	24.1	20.3	38.0	12.3	22.4	76.7	71.1
Ga (ng/g)	36.0	22.7	19.8	37.2	37.4	43.3	42.9	<MDL	<MDL	<MDL	<MDL	0.3	0.1	1.6	2.5
Hg (ng/g) *	1.0	1.1	0.9	2.1	2.0	1.9	2.2	1.4	1.2	1.4	1.5	0.8	1.0	0.4	0.5
Trees 4 & 5:	1.9 (150 cm) 0.8 (77 cm)			2.4 (55 cm) 0.6 (52 cm)			- -			- -			0.3 (68 cm) 0.6 (77 cm)		
Mg (µg/g)	254	1079	321	95.9	104	98.2	359	122	86	105.6	74.2	98.2	122.4	319.8	179.3
Mn (µg/g)	55.1	42.1	29.4	18.0	59.6	30.7	53.0	49.7	17.6	45.3	59.7	64.4	37.8	74.7	71.6
Ni (µg/g)	3.3	6.3	4.9	1.9	0.43	2.7	0.71	1.2	0.5	0.5	0.5	0.4	0.7	5.0	1.2
Pb (µg/g)	0.48	5.3	0.17	0.53	0.07	0.67	<MDL	<MDL	<MDL	<MDL	<MDL	<MDL	<MDL	<MDL	<MDL
Sr (µg/g)	52.6	20.4	17.9	18.1	11.1	12.6	10.4	6.7	6.0	5.7	8.9	2.6	44.6	18.2	29.6
Th (ng/g)	<MDL	<MDL	<MDL	<MDL	<MDL	<MDL	<MDL	<MDL	<MDL	<MDL	<MDL	<MDL	9.4	<MDL	<MDL
U (ng/g)	<MDL	<MDL	<MDL	<MDL	<MDL	<MDL	<MDL	<MDL	<MDL	<MDL	<MDL	<MDL	<MDL	<MDL	<MDL

* Hg was determined by DMA in 5 trees per species, except for *Pinus Echinata* and *Pinus Taeda* (*n* = 3). Tree circumference (cm) is given in row 3 for trees 1–3 and in parentheses next to their Hg concentration value for trees 4 and 5.

**Table 4:** Direct mercury analyzer (DMA) and ICP-MS instrumental settings.

**DMA**	
Gas flow	200 mL/min
Drying	200 °C for 60 s
Decomposition	650 °C for 180 s
Purge	60 s
Amalgamator heat	900 °C for 12s
Record	60 s
**Plasma Parameters**	
Cool gas flow	14 L/min
Auxiliary gas flow	0.9 L/min
Sample gas flow	1.1 L/min
RF power	1280 W
**ICP-MS Data Acquisition**	
Isotopes in LR	^9^Be, ^85^Rb, ^88^Sr, ^107^Ag, ^111^Cd, ^133^Cs, ^137^Ba, ^205^Tl, ^208^Pb, ^232^Th, ^238^U
Isotopes in MR	^24^Mg,^51^V, ^52^Cr, ^55^Mn, ^56^Fe, ^59^Co, ^60^Ni, ^63^Cu, ^69^Ga
Integration time	10 ms (LR); 50 ms (MR)
Mass window	20% for LR; 125% for MR
Points per peak	50 (LR); 20 (MR)
Runs/passes	3/3 (E-scan)

LR = Low Resolution; MR = Medium Resolution.
